# Experiments and Observations on Certain Sensations of the Eye, Connected with the Theory of Vision

**Published:** 1799-10

**Authors:** C. R. Aikin

**Affiliations:** Surgeon


### *Experiments and Observations on certain Sensations of the Eye, connected with the Theory of Vision: By* C. R. AIKIN, *Surgeon.*

IT is a well-known fact that, in certain circumstances, impressions made on the retina of the eye, totally distinct from those of light, will, nevertheless, excite the sensation of vision. Thus a blow upon the eye will produce the sensation of a flash of light, which will be the most vivid even when the eye is shut, or in perfect darkness. So in many diseases of the eye, as in inflammation, or in the incipient state of amaurosis, a number of irregular sensations of light are perceived, sometimes like momentary flashes, at other times like minute insects passing over the field of vision, which continue either till the eye is restored to a healthy state, or till the disease has rendered the retina incapable of transmitting any impression whatever.

This aptitude in the retina to transmit the idea of light from other impressions has been frequently noticed, but I have not seen it observed, that these sensations correspond in other respects with some of the known laws of vision, especially with regard to the apparent place of the image and its intensity.

This will be illustrated by the following experiments; but I must premise that in these, as in all experiments upon the minuter sensations of the eye, some practice, and much attention is necessary, in order to detect these transitory perceptions, which, nevertheless, are in themselves perfectly real.

EXPE-

### Experiment first.

Make a moderate preſſure upon the upper eye-lid at the external angle of the eye, ſo as to compreſs the globe of the eye as far back as the bones of the orbit will permit. The preſſure may be made with the little finger, but it is better done with the blunt head of a probe, or a ſimilar inſtrument, in order that the preſſure may be diſtinct, and occupy only a ſmall, well-defined ſpace. If then the attention be directed towards the noſe, a ſmall dark ſpot will be perceived, apparently upon the noſe, and exactly on the oppoſite ſide of the eye to that where the preſſure is made. If the preſſure be now carried to different parts of the globe of the eye, the ſame ſpot will accompany the preſſure, and always in the ſame direction; that is, it will appear above when the lower part is preſſed, and below when the upper.

The exact appearance of this image, when the eye is open, is, to my eyes, that of a dark ſpot, ſurrounded with a faint ring of light. This is more viſible when a piece of black or white paper is laid upon the noſe, over the apparent place of the ſpot, for a reaſon to be afterwards mentioned. The ſame image appears when the eye is ſhut, only then it is more illuminated and better defined. It is difficult to produce the image on the upper part of the eye without giving ſome pain, owing to the greater quantity of fat covering the under eye-lid (where the preſſure muſt be made), which in ſome degree defends the eye from any local preſſure.

### Experiment Second.

Let the preſſure be made on the external angle of both eyes, at the ſame time, whilſt the ſight is directed ſtraight forwards, and downwards to any object, at the neareſt focus of diſtinct viſion. If attention be then paid to the noſe it will appear double, with the image of a black ſpot upon each; that belonging to the *right noſe* (if I may ſo ſpeak) being produced by the preſſure of the left eye, and *vice verſa,* which may be directly proved by diſcontinuing the preſſure on either ſide alternately.

### Experiment Third.

Let the eye-lid of one eye be cloſed, and the preſſure be made upon that eye in the manner above-mentioned, and the image will appear in the ſame relative ſituation. Then carry the probe, with a gentle preſſure, to the anterior part of the eye (which could not be done when the eye was open), and the ſpot will be found to diſappear as ſoon as the preſſure is carried beyond the ciliary ligament, and no image will be formed as long as the probe remains over any part of the cornea; but, if exact attention be paid to the ſenſations, the image will return as the preſſure paſſes again over the tunica albuginea.

These experiments ſhew, in a curious manner, how an impreſſion on the retina, totally foreign to that of light, will however produce the ſame ſenſation; and this ſenſation will excite the idea of a viſible object in the ſame place in which it would be ſituated if it really were the cauſe of the ſenſation, that is, in any part of a right line projected from the ſentient point of the retina, and paſſing through the center of the eye to any part of external viſible ſpace. That in the above experiments, the ſenſation is produced immediately on the point expoſed to preſſure, is ſhewn from the diſappearance of the image as ſoon as the preſſure is removed to the cornea, a part ſituated beyond the inſertion of the retina. There exiſts this difference between the artificial ſenſation of viſion and real ſight, that in the latter the impreſſion on the retina, which is the immediate cauſe of ſenſation, is actually produced through the medium of an external object; whereas in the former, the impreſſion is independant of a viſible object, and excites the idea of one that does not exiſt. Hence, in the caſe of the above experiments, the more completely the effect of real external object is excluded, the more vividly will the idea of it be artifically excited; and this is brought about either by cloſing the eyelid, or by placing any uniform ſurface, ſuch as black or white paper, in the ſituation which an external object would occupy, in order to excite its image on that part of the retina on which the artificial impreſſion is made.

In an ingenious paper by Mr. Elliott, a very accurate deſcription is given of the effects of conſiderable preſſure made upon the whole globe of the eye, by applying the open hand over the whole of the anterior part of the eye. I have once or twice repeated the experiment, and with nearly the ſame ſenſations which he deſcribes, but it is a painful, and I think not a very ſafe experiment. One or two circumſtances in it deſerve notice.

The preſſure is made by laying the hand upon the eye, and keeping up a a firm long-continued force, which, after a while, produces a circular luminous ſpectrum within the eye, ſomewhat reſembling a full moon, with a double halo around it; and if the preſſure be longer continued, the ſpectrum grows fainter, and gradually diſappears, leaving the eye for a while inſenſible to external objects. Now, as the preſſure is only made upon the cornea in this painful experiment, and therefore at a diſtance from the retina, it follows that the ſenſation of light is produced from the communication of impreſſion from the anterior to the poſterior part of the globe, and this accounts for the ſpectrum not being *immediately* produced, and the great degree of force required to produce it at all; whereas in the experiments which I have given, the part of the eye immediately preſſed is that which excites the ſenſation, as is proved by the apparent place of the ſpectrum; its its requiring but little force of preffure, and not being produced upon any part of the cornea.

Another circumftance in which this artificial fenfation of light correfponds with real vifion is, in the increafed intenfity and diftinctnefs of the perception, in proportion as it is excited nearer to the center of the retina, which point is well known to be that of the moft diftinct vifion. To illuftrate this, let the following experiment be made:

EXPERIMENT FOURTH.

Let preffure be made, as in the foregoing experiments, upon the external angle of one eye, as far back as the bones of the orbit will allow, and with the eye-lid open, and the fight directed ftraight forwards; this, as before-mentioned, will throw the image of a dark fpot upon the nofe. Then, without difcontinuing the preffure, let the eye, by rolling inwards, be directed towards the fpot, and it will appear to diminifh in fize, become more diftinct, and in fome degree to retire from the eye, which will, however, get nearer to the fpot, and almoft touch it, as it were, but not quite: then if the eye be gradually rolled outwards, the fpot will as gradually become lefs diftinct, will enlarge, and by enlarging will appear to purfue the eye for a little way, as it appeared before to retire from the eye by diminifhing.

This experiment is explained by reflecting that, as the preffure is all along continued, when the eye rolls inward, the parts of the retina fucceffively prefented to the outward preffure, are conftantly approaching to the central point of the retina, where vifion is the moft diftinct, and therefore the apparent object (the black fpot) is continually getting fmaller and more defined; but it can never exactly coincide with the axis of the eye, becaufe the eye, however prominent, can never be rolled fo far inwards as to prefent the central point of the retina to any external preffure.

Experiments of this kind may fuggeft feveral very curious queftions, with regard to the quality of the nerves belonging to the organs of fenfe, of tranfmitting only their peculiar fenfations, and it would be an interefting queftion to folve—Whether the retina acquires by habit this quality of referring various impreffions to the fenfation of vifion; or whether this is an inherent quality of this organ?

In either cafe, the fact appears to me certain, that thefe artificial fenfations follow the laws of real vifion, both with refpect to the apparent fituation of the object, and in being more eafily excited on the parts of the retina the moft fufceptible to light.

As

As the optic nerve, even where it penerates the coats of the eye, is not ſuſceptible of the external impreſſion of light, ſo we may probably conclude that no kind of impreſſion which was confined to the nerve alone, and not conveyed by the medium of the retina, would excite the idea of light; and perhaps this may be the reaſon why in ſome caſes of fever affecting the brain, in phrenitis, and in general, in all caſes where the brain, or its membranes, acquire an unuſual degree of irritability, the ſymptom of deep-ſeated pain of the forehead, ſhooting into the eye-ball, precedes that of increaſed ſuſceptibility to light, owing, as I conceive, to the morbid irritability being communicated gradually down the optic nerve to the retina, and that it is only when the latter is affected that light becomes painful.

The other organs of ſenſe are not ſo eaſily made the ſubject of experiment as the eye, but they appear in ſome degree to poſſeſs the ſame property of tranſmitting their peculiar ſenſations from very various kinds of impreſſions. Thus the ſenſation of ſinging in the ear, which often occurs during a ſevere cold, may be occaſioned by an increaſed irritability of the membrane of the tympanum, communicated along the Euſtachian tube. The ſame irregular ſenſations of ſound follow a violent blow on the external ear, and attend incipient deafneſs, in the ſame manner as thoſe of the light attend the eye in ſimilar circumſtances; and in many caſes of general affection of the head, where light is painful, noiſe is equally ſo. Perhaps the ſtrong ſenſation of taſte made on the tongue, by the metallic influence in the Galvanic experiments, may be owing to the ſame cauſe, and not to an actual ſolution of any part of the metal in the ſaliva, which muſt otherwiſe be imagined.

